# Supplementary material for: Singular effect of linkage on long-term genetic gain in Fisher’s infinitesimal model
Source: PNAS Nexus. 2024 Jul 26;3(8):pgae314. doi: 10.1093/pnasnexus/pgae314 (PMC11316219; doi:10.1093/pnasnexus/pgae314)
Supplement: pgae314_Supplementary_Data [file pgae314_supplementary_data.zip › PNASNEXUS-PNASNEXUS-2024-00309RR-s05.pdf]

Supplementary Information for

Singular effect of linkage on long-term genetic gain in Fisher's infinitesimal model.

Elise Tourrette and Olivier C. Martin

Corresponding author: Olivier C. Martin
Email: olivier.c.martin@inrae.fr

This PDF file includes:

Supplementary text
Legends for Datasets S1 to S2

Other supplementary materials for this manuscript include the following:

Datasets S1 to S2

Supplementary Information Text

APPENDIX I: Fourier series of a recombined gamete

Here we determine the coefficients of the Fourier series of a recombined gamete's profile as a function of those of its two constitutive gametes and of the angle ψ specifying the position of the crossover P_1 as represented in Fig.1 of the Main. We begin with the periodic step (Heaviside) function that is 1 in the intervals $]2n\pi, (2n+1)\pi[$ and vanishes otherwise. Its Fourier series representation is:

$$H(\theta) = \frac{1}{2} + \frac{2}{\pi} \sum_{k=0}^{k=\infty} \frac{1}{(2k+1)} \sin\left[(2k+1)\theta\right]$$

Let the two constitutive gametes in Fig. 1 of the Main have the profiles $x(\theta)$ and $x'(\theta)$. Each of these profiles has a Fourier series representation as given in Eq.3 of the Main; let the corresponding coefficients be X_k and X'_k. The recombined gamete's profile is then $x''(\theta) = x(\theta)H(\theta-\psi) + x'(\theta)H(\theta-\psi-\pi)$. Since this is linear in x and x', the Fourier coefficients of a recombined gamete are linear in the Fourier coefficients of its constitutive gametes. Consider for instance the contribution of the first constitutive gamete using its series representation:

$$x(\theta)H(\theta-\psi) = \left[\sum_{-\infty}^{+\infty} e^{ik\theta} X_k\right] \left[\frac{1}{2} + \frac{-i}{\pi} \sum_{-\infty}^{+\infty} \frac{e^{i(2k+1)(\theta-\psi)}}{2k+1}\right]$$

Carrying out this product and extracting the coefficient of $\exp(ik\theta)$ gives the kth Fourier coefficient for that first constitutive gamete's contribution to the recombined one:

$$\frac{X_k}{2} + \frac{-i}{\pi} \sum_{-\infty}^{+\infty} \frac{X_{k-(2k'+1)}}{2k'+1} e^{-i(2k'+1)\psi}$$

The contribution from the second constitutive gamete is obtained similarly after the replacement of X by X' and of ψ by $\psi+\pi$. Adding the contributions of both of these, we obtain that the kth Fourier coefficient of the series for the recombined gamete is:

$$\begin{aligned} X''_k = \; & \frac{X_k + X'_k}{2} + \frac{-i}{\pi} \sum_{-\infty}^{+\infty} \frac{X_{k-(2k'+1)}}{2k'+1} e^{-i(2k'+1)\psi} \\ & + \frac{i}{\pi} \sum_{-\infty}^{+\infty} \frac{X'_{k-(2k'+1)}}{2k'+1} e^{-i(2k'+1)\psi} \end{aligned}$$

A consequence of this linear relation is that at all generations the distribution of Fourier coefficients – conditional on the different ψ angles – remains multi-dimensional complex normal since their initial distribution is so.

APPENDIX II: Recursions for the variances of the Fourier coefficients

As explained in the main part of the paper, because of the rotational invariance of the distribution of profiles ($\theta \to \theta + \delta$), the (complex) covariance matrix of the Fourier coefficients (Eq.4 of the Main) is diagonal. Since X''_k is a linear combination of independent random variables, its (complex) variance is just the sum of the complex variances of those variables. Let us first calculate the expectation of $\overline{X''_k} X''_k$:

$$\begin{aligned} E\left[\overline{X''_k} X''_k\right] = \; & \frac{E\left[\overline{X_k} X_k\right] + E\left[\overline{X'_k} X'_k\right]}{4} \\ & + \frac{1}{\pi^2} \sum_{-\infty}^{+\infty} \frac{\left[\overline{X_{k-(2k'+1)}} X_{k-(2k'+1)}\right]}{(2k'+1)^2} \\ & + \frac{1}{\pi^2} \sum_{-\infty}^{+\infty} \frac{\left[\overline{X'_{k-(2k'+1)}} X'_{k-(2k'+1}\right]}{(2k'+1)^2} \end{aligned}$$

In the notation of the main part of the paper, this leads to the recursion when going from one generation to the next:

$$\sigma^2_{k,g+1} = \frac{\sigma^2_{k,g}}{2} + \frac{2}{\pi^2} \sum_{-\infty}^{+\infty} \frac{\sigma^2_{k-(2k'+1),g}}{(2k'+1)^2}$$

Using the diffusion interpretation presented in the main part of the paper, we see that half of the matter at each site is left in place while the other half is shared across other sites according to a convolution kernel decaying inversely with the square distance on the line of (odd) integers. This convolution kernel is normalized to 1 as it should be because of the result:

$$\frac{4}{\pi^2} \sum_{-\infty}^{+\infty} \frac{1}{(2k'+1)^2} = 1$$

In the context of cycles where one first selects gametes having a given value for G, the $k=0$ Fourier component for all gametes are first set to that value before one proceeds with the mating. This then sets the corresponding variance to 0 and so, in the recursion formula given above, $\sigma^2_{k=0,g}$ must be replaced by 0, and then one recovers Eq.6 of the Main.

Dataset S1 (separate file).

R script to implement the recurrences numerically.

Dataset S2 (separate file).

R script to analyze the numerical results and produce figures 2 and 3 of the Main.

© The Author 2024. Published by Oxford University Press. All rights reserved. For permissions, please e-mail: journals.permissions@oup.com
